# Physical activity and cancer-related cognitive impairment: Is physical activity associated with perceived difficulties and unmet needs?

**DOI:** 10.1007/s00520-026-11429-9

**Published:** 2026-11-07

**Authors:** Darren Haywood, James Murray, Francesco Bettariga, Oscar Lederman, Alanah Pike, Evan Dauer, Jordan Curry, Dominic O’Connor, Nicolas H. Hart

**Affiliations:** 1 Human Performance Research Centre, INSIGHT Research Institute, School of Sport, Exercise and Rehabilitation, Faculty of Health, University of Technology Sydney (UTS), Moore Park, Sydney, NSW 2030 Australia; 2 Department of Mental Health, St Vincent’s Hospital Melbourne, Fitzroy, VIC Australia; 3 Centre for Mental Health and Brain Sciences, Swinburne University of Technology, Hawthorn, Australia; 4 School of Population Health, Faculty of Health Sciences, Curtin University, Bentley, WA Australia; 5 Exercise Medicine Research Institute, School of Medical and Health Science, Edith Cowan University, Cowan, WA Australia; 6 Exercise & Social Equity (NExuS) Research Group, Discipline of Psychiatry and Mental Health, School of Clinical Medicine, University of New South Wales, Sydney, NSW 2052 Australia; 7 Wolfson Palliative Care Research Centre, Hull York Medical School, University of Hull, Cottingham Road, Hull, UK; 8 Department of Health and Exercise Science, Colorado State University, Fort Collins, CO USA; 9 School of Health Sciences, University of Nottingham, Nottingham, UK; 10 Caring Futures Institute, College of Nursing and Health Sciences, Flinders University, Adelaide, SA Australia; 11 Cancer and Palliative Care Outcomes Centre, Faculty of Health, Queensland University of Technology (QUT), Brisbane, QLD Australia; 12 Institute for Health Research, University of Notre Dame Australia, Fremantle, WA Australia

**Keywords:** Cancer-related cognitive impairment, Physical activity, Exercise, Unmet needs, Cognition

## Abstract

**Purpose:**

Cancer-related cognitive impairment (CRCI) is associated with significant functional difficulties and unmet needs. Physical activity (PA) is a promising approach for reducing perceived CRCI; however, its relationship with perceived CRCI-related difficulties and unmet needs remains unclear. This study examined the associations between PA, perceived cognitive functioning, and perceived CRCI-related difficulties and unmet needs, and assessed whether PA explained additional variance in perceived CRCI-related difficulties and unmet needs after controlling for perceived cognitive functioning.

**Methods:**

In this cross-sectional study, adult cancer survivors receiving curative-intent treatment (*n = *418) who self-identified as experiencing CRCI completed the PROMIS-Cog, MASCC COG-IMPACT, and modified Godin-Shephard Leisure-Time Physical Activity Questionnaire. Bivariate correlation analyses and hierarchical multiple regression analyses were conducted to examine associations between physical activity, perceived cognitive functioning, and CRCI-related difficulties and unmet needs, and to determine whether physical activity explained additional variance in CRCI-related difficulties and unmet needs beyond perceived cognitive functioning.

**Results:**

Greater engagement in light-, vigorous-, and resistance-based PA was associated with better perceived cognitive functioning (*r* = 0.10–0.14; *p* < 0.05) with small effects. PA was also associated with fewer psychological (*r* =− 0.11; *p*  < 0.05) and verbal communication (*r* =− 0.13; *p*  < 0.05) CRCI-related difficulties, but more CRCI-related informational need difficulties (*r* = 0.13–0.17; *p*  < 0.01) with small effects. PA was not associated with CRCI-related unmet needs (*p* > 0.05). After controlling for perceived cognitive functioning, greater PA was associated with greater perceived CRCI-related difficulties and unmet needs in several domains (*p* < 0.05).

**Conclusions:**

Physical activity was associated with reduced perceived CRCI severity, supporting its recognition as a promising supportive care strategy. However, associations between greater physical activity participation and lower levels of perceived CRCI may not correspond with lower levels of CRCI-related difficulties and unmet needs. PA-based CRCI interventions should be embedded within holistic survivorship care models that proactively identify and address perceived CRCI-related difficulties and unmet supportive care needs.

**Supplementary Information:**

The online version contains supplementary material available at https://doi.org/10.1007/s00520-026-11429-9.

## Introduction

Cancer-related cognitive impairment (CRCI) is a common consequence of cancer, its treatments, and associated psychosocial impacts, characterised by perceived and/or objectively measured impairments in cognitive domains of attention, processing speed, executive function, and memory [1–3]. These cognitive changes can emerge and persist across any stage of the cancer continuum and are increasingly recognised as a clinical priority due to their effects on daily functioning and quality of life [1–6]. Large-scale symptom screening data across diverse cancer populations indicate that cognitive symptoms are present in more than two-thirds of cancer survivors, with approximately one-third reporting moderate-to-severe cognitive concerns [7, 8]. There is also heterogeneity in CRCI trajectories and mechanisms, and these cognitive symptoms often co-occur with other survivorship challenges (e.g., fatigue, sleep quantity and quality, and psychological challenges), emphasising the need for holistic supportive care approaches [4, 8–11].

Perceived cognitive functioning in cancer survivors often shows limited concordance with objective neuropsychological performance [12, 13]. However, perceived cognitive functioning is more strongly associated with functional outcomes and quality of life when compared to objective assessments in cancer survivors reporting CRCI [14, 15]. Qualitative evidence further shows that cancer survivors who perceive cognitive functioning changes after a cancer diagnosis describe these impairments as strongly impacting confidence and identity, occupational functioning, relationships, and psychological well-being, all of which are central to quality of life [16]. Accordingly, perceived cognitive functioning is a key component of cognitive functioning assessments in cancer survivors and represents a primary target for intervention.

Physical activity, defined as any bodily movement produced by skeletal muscles resulting in energy expenditure [17], is a widely recommended, non-pharmacological supportive care strategy across the cancer continuum with demonstrated efficacy across a wide range of outcomes [18–23]. International guidelines support the use of aerobic and resistance activities to improve physical function, quality of life, common treatment-related symptoms, and other clinical outcomes [24–26]. Evidence suggests physical activity is a cost-effective, feasible, and accessible approach to mitigate CRCI (i.e., improve impaired cognitive functioning), with recent umbrella reviews suggesting both aerobic and resistance activities improve perceived cognitive functioning in cancer survivors [27, 28]. Proposed mechanisms of action include improved cerebral perfusion, reductions in inflammation and fatigue, and indirect benefits through improved mood and sleep [29, 30]. While this is an area of significant interest regarding its utility in reducing CRCI, it remains unclear how physical activity relates to the perceived difficulties and unmet supportive care needs that arise from CRCI.

Perceived daily living and psychosocial difficulties resulting from CRCI, and their associated unmet supportive care needs, are key determinants of social and occupational participation and quality of life [4, 10, 31–33]. Addressing perceived CRCI-related difficulties and unmet supportive care needs is vital to providing high-quality person-centred care that promotes wellbeing, functioning, and participation [31, 32, 34]. While it is consistently found that a lesser severity of perceived cognitive impairment is associated with fewer CRCI-related difficulties and unmet needs [4, 31, 34], physical activity may influence these outcomes through mechanisms beyond cognitive impairment severity. Speculatively, physical activity may mitigate the impact of CRCI on daily life by improving physical functioning and broader psychosocial resources (i.e., resilience, self-efficacy) [35, 36], thereby reducing CRCI-related difficulties and related unmet supportive care needs through compensation of cognitive limitations. However, individuals who engage in higher levels of routine physical activity and healthy lifestyle behaviours often demonstrate greater health literacy and information seeking, place a stronger priority on health and functioning, and exhibit heightened bodily, psychological, and cognitive awareness [36–38]. These characteristics may, paradoxically, be associated with greater perceived CRCI-related difficulties and unmet supportive care needs, as increased knowledge, prioritisation, and awareness of self and health may heighten their sensitivity to cognitive changes and foster stronger expectations regarding optimal functioning and access to supportive care. Further, more physically active cancer survivors may spend more time in cognitively, physically, and socially demanding contexts or roles (e.g., returning to work, community participation, group exercise) where cognitive limitations be more salient, potentially increasing perceived difficulties even if cognitive functioning is greater than that of their less active counterparts [34, 39, 40]. Therefore, associations between greater physical activity participation and lower levels of perceived CRCI severity may not correspond with lower levels of CRCI-related difficulties and unmet needs.

A clearer understanding of the relationships between physical activity, perceived cognitive function, perceived CRCI-related difficulties, and unmet needs is needed to inform holistic, person-centred care and optimise survivorship support pathways. Should perceived CRCI-related difficulties and unmet needs remain associated with physical activity participation beyond the contribution of perceived cognitive functioning, this would underscore the need for clinicians and interventionists to provide appropriate, targeted support [9]. Accordingly, the aims of this study were to (1) examine associations between physical activity, perceived cognitive functioning, and perceived CRCI-related difficulties and unmet supportive care needs among cancer survivors, and (2) assess if physical activity explains additional variance in perceived CRCI-related difficulties and unmet supportive care needs after accounting for perceived cognitive functioning.

## Methods

### Design

A cross-sectional self-report study design was used. Ethics approval was obtained from the University of Technology Sydney Medical Research Ethics Committee (ETH25-11,032), and participants provided informed consent prior to participating. This study was reported in accordance with the Strengthening the Reporting of Observational Studies in Epidemiology (STROBE) guidelines [41], and a completed STROBE checklist is provided as Supplementary Material.

### Participants

Participants were adult cancer survivors, defined as: 18 years or older, with a current diagnosis of cancer (any type), currently receiving treatment with curative intent, self-identifying as experiencing CRCI, and fluent in English (reading and speaking). The sole exclusion criterion was a diagnosis of another neurocognitive or neurological disorder.

Participants were recruited based on their self-identification of CRCI, rather than on whether they met predefined cognitive assessment cut-off scores. This approach was adopted in recognition of qualitative evidence demonstrating that survivors’ perceived cognitive *changes* relative to their pre-diagnosis functioning are central to their lived experience and closely linked to perceived CRCI-related difficulties and unmet supportive care needs [16].

### Recruitment

Participants were recruited via Prolific [42], a well-established online research participant platform. Prolific has consistently been shown to provide valid and reliable data and is widely used in neuropsychological research, including studies in oncology populations [31, 43–46]. Participants were reimbursed according to Prolific’s recommended rates.

### Measures

As a part of a larger study, participants completed a self-report survey that included demographic and clinical questions (e.g., age, sex, employment status, cancer type, treatments received, etc.), the Multinational Association of Supportive Care in Cancer – Unmet Needs Assessment of Cancer-Related Cognitive Impairment Impact (MASCC COG-IMPACT) tool [31], the Patient-Reported Outcomes Measurement Information System-Cognitive Function Scale 8a (PROMIS-Cog) [47], and a Modified Godin-Shephard Leisure-Time Questionnaire (GSLTPAQ) [48, 49].

The MASCC COG-IMPACT is a validated 55-item self-report measure assessing perceived CRCI-related difficulties and unmet supportive care needs over the last month across eight subscales: Executing Regular Activities, Finding Meaning and Enjoyment in Activities, Relational Difficulties, Occupational/Vocational Functioning, Psychological Challenges, Verbal Communication Challenges, Social Functioning and Withdrawal, and Informational Needs. A perceived CRCI-related score for (a) difficulties and (b) unmet needs can be calculated for each MASCC COG-IMPACT subscale [31]. Difficulties subscale scores range from 0 to 1, and unmet needs subscale scores range from 0 to 3 – greater scores relate to greater levels of difficulties and unmet needs, respectively. The MASCC COG-IMPACT also has cut-off scores for the determination of no unmet needs (subscale score = 0), low unmet needs (subscale score < 1), low-to-moderate unmet needs (subscale score ≥ 1), moderate-to-high unmet needs (subscale score ≥ 2), and high unmet needs (subscale score = 3) [31, 32].

The PROMIS-Cog 8a [47] is an eight-item validated self-report assessment of perceived cognitive function over the previous seven days. The PROMIS-Cog is recommended by the Neuroscience Initiative Working Group to be used for CRCI research [50, 51] and provides an overall *T*-score across its items, ranging from 22.41 to 63.48, with higher scores reflecting better cognitive functioning and 50 representing a normative mean score. The PROMIS-Cog also has a cut-off for clinically meaningful perceived cognitive impairment for cancer survivors (raw score < 34) [50, 51].

Physical activity was measured using an adapted form of the Godin-Shephard Leisure-Time Physical Activity Questionnaire (GSLTPAQ) [48, 49]. This self-reported measure includes three core items assessing how often participants engaged in light-, moderate-, and vigorous-intensity physical activity lasting at least 15 minutes over the prior seven days. For each intensity category, examples of common aerobic activities are provided to support accurate recall. As per Umlauff et al. [49], an additional item was incorporated to capture the frequency and duration of resistance physical activity. The GSLTPAQ facilitates the calculation of a weighted weekly moderate-to-vigorous physical activity (MVPA) in minutes through the following formula: weighted MVPA = 2 × (frequency of vigorous physical activity × duration of vigorous physical activity) + (frequency of moderate physical activity × duration of moderate physical activity). This further allows the classification of participants meeting, or not meeting, weighted MVPA aerobic guidelines for cancer survivors (≥ 150 min per week) [52]. A total weekly duration of resistance-based exercise can also be calculated (resistance-based frequency × duration).

### Analysis

Descriptive and frequency analyses were used to characterise the sample via means, standard deviations, counts, ranges and percentages; and correlational analyses were used to examine associations between physical activity, perceived cognitive functioning, and perceived CRCI-related difficulties and unmet needs among cancer survivors via Pearson’s bivariate correlation analyses (for continuous variables and ordinal variables) and point-biserial correlation analyses (for binary variables). The strength of bivariate correlations was interpreted as small *r* < 0.3, medium *r* = 0.31 to *r* = 0.69, and large r ≥ 0.70 [53].

To assess if physical activity explains additional variance in perceived CRCI-related difficulties and unmet needs after accounting for perceived cognitive functioning, 16 hierarchical (two-step) multiple regression analyses were used (one per (a) difficulties, and (b) unmet needs indices of each of the eight MASCC COG-IMPACT subscales). The criterion variables were the MASCC COG-IMPACT difficulties and unmet needs subscales, and the predictor variables were the PROMIS-Cog *T*-score, weighted MVPA, and resistance-based activity weekly duration. Weighted MVPA was chosen to represent aerobic activity as it is a commonly operationalised aerobic activity metric in survivorship research, aligns with clinical exercise guidelines, and has shown consistent associations with improvements in cancer survivorship outcomes [54]. The PROMIS-Cog was entered on step 1 of the regressions, while weighted MVPA and resistance-based activity weekly duration were entered on step 2. This facilitated the examination of the utility of weighted MVPA and resistance-based activity (collectively and uniquely) to account for perceived CRCI-related difficulties and unmet needs beyond perceived cognitive functioning.

To account for testing of multiple models, *p*-values associated with the F-change of step 2 of each regression model were adjusted using the Benjamini–Hochberg False Discovery Rate (FDR) procedure, controlling the expected proportion of false positives at α = 0.05 [55]. Overall regression model *p*-values of < 0.001 were treated as *p* = 0.001 for the FDR procedure. A standard 0.05 alpha level was used for the assessment of the overall model, which is not associated with our hypothesis testing.

## Results

### Participants

Four hundred and twenty-two (*n* = 422) cancer survivors completed the survey, with three removed due to failing 50% or more of the four attention check items. Thus, a total sample of 418 cancer survivors was used for this study. Sample characteristics are reported in Table 1. Although an a priori sample size calculation was not performed, the final sample of 418 participants substantially exceeded commonly cited sample size recommendations for multiple regression analyses. For example, Green (1991) recommends a minimum sample size of 74 participants for testing an overall model and 107 participants for testing individual predictors in a three-predictor regression model. Further, a Benjamini–Hochberg FDR adjustment was used to test the incremental change in accounted variance. Accordingly, the present study was well above conventional recommendations for regression-based analyses.

**Table 1:** Sample characteristics (*N = *418)

Characteristic	Mean (SD)/count
Age (years)	39.4 years (14.8); min. 19 years. Max. 78 years
Sex at birth	
Male	129 (30.9%)
Female	289 (69.1%)
Ethnicity	
Caucasian	155 (37.1%)
African/African American	227 (54.3%)
Asian	10 (2.4%)
Hispanic or Latino	6 (1.4%)
Native American/American Indian	3 (0.7%)
Other	17 (4.1%)
Primary cancer type	
Breast	167 (40.0%)
Prostate	46 (11.0%)
Bowel/colorectal	17 (4.1%)
Melanoma	15 (3.6%)
Lung	34 (8.1%)
Lymphoma	13 (3.1%)
Leukaemia	21 (5.0%)
Brain	27 (6.5%)
Pancreatic	4 (1.0%)
Myeloma	1 (0.2%)
Cervical	13 (3.1%)
Thyroid	9 (2.2%)
Testicular	4 (1.0%)
Ovarian	9 (2.2%)
Sarcoma	8 (1.9%)
Other	21 (5.0%)
Country of residence	
South Africa	230 (55.0%)
United States of America	74 (17.7%)
United Kingdom	62 (14.8%)
Others	52 (12.4%)
Treatments receiving	
Chemotherapy	208 (49.8%)
Radiation	120 (28.7%)
Hormone treatment	96 (23.0%)
Targeted therapies	69 (16.5%)
Surgery	76 (18.2%)
Other	63 (15.1%)

Rounding and multiple treatment modalities may result in percentages not equalling 100%. Treatment categories were not mutually exclusive. Surgery refers to surgery that had either been recently received or was planned at the time of participation

### Key variable descriptive and frequencies

Descriptive and frequency statistics for key variables across perceived CRCI-related difficulties and unmet needs, cognitive functioning, and physical activity are presented in Table 2. The greatest CRCI-related difficulties were perceived for Psychological Challenges, Social Functioning and Withdrawal, and Finding Meaning and Enjoyment in Activities, while the greatest perceived CRCI-related unmet needs were reported in Psychological Challenges, Informational Needs, and Finding Meaning and Enjoyment in Activities. As expected, mean perceived cognitive function (*T*-score = 40.7) was notably lower than the established norm (*T*-score = 50.0), whereby 84.7% of participants displayed clinically meaningful perceived cognitive impairment according to the cancer-specific cut-off score [50, 51]. Most physical activities completed by people were self-assessed as light-to-moderate-intensity aerobic activities, with 68.4% of participants meeting cancer survivor weighted MVPA per week guidelines.

**Table 2:** Descriptive and frequency statistics for key variables (*N = *418)

Variable	Mean (SD)/count	Range (min–max)	Cut-off classifications N (%)
MASCC COG-IMPACT			
Executing regular activities			
*Difficulties*	0.64 (0.28)	0–1	-
*Unmet needs*	0.71 (0.78)	0–3	No unmet needs: 114 (27.3%) Low unmet needs: 178 (42.6%) Low-to-moderate unmet needs: 81 (19.4%) Moderate-to-High unmet needs: 40 (9.6%) High unmet needs: 5 (1.2%)
Finding meaning and enjoyment in activities			
*Difficulties*	0.68 (0.35)	0–1	-
*Unmet needs*	0.79 (0.88)	0–3	No unmet needs: 160 (38.3%) Low unmet needs: 104 (24.9%) Low-to-moderate unmet needs: 81 (19.4%) Moderate-to-High unmet needs: 63 (15.1%) High unmet needs: 10 (2.4%)
Relational difficulties			
*Difficulties*	0.53 (0.33)	0–1	-
*Unmet needs*	0.66 (0.78)	0–3	No unmet needs:142 (34.0%) Low unmet needs: 158 (37.8%) Low-to-moderate unmet needs: 82 (19.6%) Moderate-to-High unmet needs: 30 (7.2%) High unmet needs: 6 (1.4%)
Occupational/vocational functioning			
*Difficulties*	0.55 (0.33)	0–1	-
*Unmet needs*	0.64 (0.76)	0–3	No unmet needs: 130 (31.1%) Low unmet needs: 174 (41.6%) Low-to-moderate unmet needs: 75 (17.9%) Moderate-to-High unmet needs: 33 (7.9%) High unmet needs: 6 (1.4%)
Psychological challenges			
*Difficulties*	0.71 (0.29)	0–1	-
*Unmet needs*	0.94 (0.89)	0–3	No unmet needs: 86 (20.6%) Low unmet needs: 164 (39.2%) Low-to-moderate unmet needs: 91 (21.8%) Moderate-to-High unmet needs: 61 (14.6%) High unmet needs: 16 (3.8%)
Verbal communication difficulties			
*Difficulties*	0.59 (0.38)	0–1	-
*Unmet needs*	0.63 (0.81)	0–3	No unmet needs: 194 (46.4%) Low unmet needs: 92 (22.0%) Low-to-moderate unmet needs: 84 (20.1%) Moderate-to-High unmet needs: 37 (8.9%) High unmet needs: 11 (2.6%)
Social functioning and withdrawal			
*Difficulties*	0.69 (0.34)	0–1	-
*Unmet needs*	0.76 (0.90)	0–3	No unmet needs: 161 (38.5%) Low unmet needs: 112 (26.8%) Low-to-moderate unmet needs: 80 (19.1%) Moderate-to-High unmet needs: 50 (12.0%) High unmet needs: 15 (3.6%)
Informational needs			
*Difficulties*	0.60 (0.35)	0–1	-
*Unmet needs*	0.83 (0.88)	0–3	No unmet needs: 126 (30.1%) Low unmet needs: 138 (33.0%) Low-to-moderate unmet needs: 90 (21.5%) Moderate-to-High unmet needs: 48 (11.5%) High unmet needs: 16 (3.8%)
PROMIS-Cog	T score 40.7 (8.0)	22.4–63.5	Clinically meaningful cognitive impairment: 354 (84.7%)
	Raw score 24.1 (8.3)	8.0–40.0	
Physical activity			
Light—mins. per week	171.5 (203.7)		-
Moderate—mins. per week	129.5 (218.0)		-
Vigorous—mins. per week	102.8 (131.8)		-
Resistance—mins. per week	120.8 (153.9)		-
Weighted MVPA	335.1 (401.2)		Weighted MVPA per week guidelines met: 286 (68.4%)

*MASCC* Multinational Association for Supportive Care in Cancer, *MVPA* moderate‑to‑vigorous physical activity, *PROMIS-Cog* Patient-Reported Outcomes Measurement Information System-Cognitive Function Scale

### Bivariate correlations

Bivariate associations are presented in Table 3. Light (*r* = 0.14*; p* < 0.01), vigorous (*r* = 0.10*; p* < 0.05), and resistance-based (*r* = 0.13*; p* < 0.01) weekly physical activity weekly duration were significantly and positively associated with perceived cognitive functioning with small effects. However, perceived cognitive functioning was not significantly associated with moderate or weighted MVPA physical activity weekly duration (*p* > 0.05). Light physical activity weekly duration was negatively associated with perceived CRCI-related psychological difficulties (*r* = –0.11; *p* < 0.05), while vigorous physical activity weekly duration was negatively associated with perceived CRCI-related verbal communication challenges difficulties (*r* = –0.13; *p* < 0.05), both with small effects. Conversely, vigorous (*r* = 0.13; *p* < 0.01), and resistance-based (*r* = 0.17; *p* < 0.001) weekly duration, as well as weighted MVPA (*r* = 0.13; *p* < 0.01), were positively associated with perceived CRCI-related informational need difficulties with small effects. Physical activity was not significantly associated with any perceived CRCI-related unmet need subdomains (*p* > 0.05). Perceived cognitive functioning was significantly negatively associated with all subdomains of CRCI-related difficulties (*r* = –0.68 to –0.48; *p* < 0.001), and unmet needs (*r* = –0.59 to –0.50; *p* < 0.001), with medium effects.

**Table 3:**
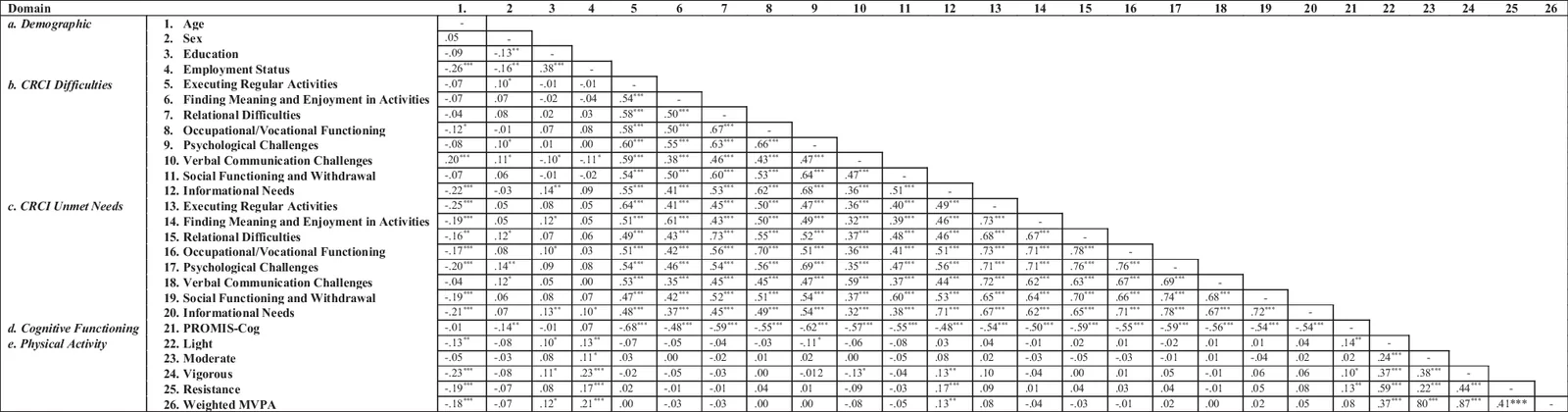
Bivariate correlation between all variables

### Multiple regression analyses

Final model results of each multiple regression analysis with CRCI-related difficulties as the criterion variables are presented in Table 4, while final results of multiple regression models with CRCI-related unmet needs as the criterion variables are presented in Table 5. The results, according to each MASCC COG-IMPACT subscale, are also described below.

**Table 4:** CRCI-related difficulties multiple regressions

Model	Unstandardised coefficients		Standardised coefficients			Correlations		
	B	Std. error	Beta	*t*	Sig	Zero-order	Partial	Part
Executing regular activities								
Cognitive impairment	−0.025	0.001	−0.697	−19.45	< 0.001	−0.027	−0.022	−0.683
Resistance PA	0.000	0.000	0.104	2.65	0.008	0.000	0.000	0.021
Weighted MVPA	8.911e-6	0.000	0.013	0.326	0.745	0.000	0.000	0.000
Finding meeting and enjoyment in activities								
Cognitive impairment	−0.021	0.002	−0.485	−11.17	< 0.001	−0.025	−0.017	−0.479
Resistance PA	0.000	0.000	0.059	1.25	0.213	0.000	0.000	−0.010
Weighted MVPA	−1.590e-5	0.000	−0.018	−0.389	0.698	0.000	0.000	−0.032
Relational difficulties								
Cognitive impairment	−0.025	0.002	−0.596	−14.93	< 0.001	−0.028	−0.021	−0.588
Resistance PA	0.000	0.000	0.072	1.65	0.100	0.000	0.000	−0.010
Weighted MVPA	−1.343e-5	0.000	−0.016	−0.374	0.708	0.000	0.000	−0.034
Occupational/vocational functioning								
Cognitive impairment	−0.023	0.002	−0.564	−13.72	< 0.001	−0.026	−0.020	−0.550
Resistance PA	0.000	0.000	0.105	2.33	0.020	0.000	0.000	0.036
Weighted MVPA	4.434e-6	0.000	0.005	0.121	0.904	0.000	0.000	0.004
Psychological challenges								
Cognitive impairment	−0.023	0.001	−0.628	−16.18	< 0.001	−0.026	−0.020	−0.616
Resistance PA	0.000	0.000	0.083	1.96	0.051	0.000	0.000	0.010
Weighted MVPA	1.109e-5	0.000	0.015	0.363	0.717	0.000	0.000	0.000
Verbal communication challenges								
Cognitive impairment	−0.027	0.002	−0.563	−13.79	< 0.001	−0.031	−0.023	−0.566
Resistance PA	−1.788e-6	0.000	−0.001	−0.016	0.987	0.000	0.000	−0.087
Weighted MVPA	−3.528e-5	0.000	−0.037	−0.839	0.402	0.000	0.000	−0.082
Social functioning and withdrawal								
Cognitive impairment	−0.023	0.002	−0.552	−13.32	< 0.001	−0.027	−0.020	−0.547
Resistance PA	0.000	0.000	0.054	1.19	0.234	0.000	0.000	−0.029
Weighted MVPA	−2.656e-5	0.000	−0.031	−0.697	0.486	0.000	0.000	−0.053
Informational needs								
Cognitive impairment	−0.022	0.002	−0.511	−12.23	< 0.001	−0.026	−0.019	−0.480
Resistance PA	0.000	0.000	0.193	4.22	< 0.001	0.000	0.001	0.165
Weighted MVPA	7.924e-5	0.000	0.091	2.01	0.045	0.000	0.000	0.130

*PA* physical activity, *MVPA* moderate‑to‑vigorous physical activity

**Table 5:** CRCI-related unmet needs multiple regressions

Model	Unstandardised coefficients		Standardised coefficients			Correlations		
	*B*	Std. error	Beta	*t*	Sig	Zero-order	Partial	Part
Executing regular activities								
Cognitive impairment	−0.054	0.004	−0.557	−13.57	< 0.001	−0.062	−0.046	−0.535
Resistance PA	0.001	0.000	0.132	2.95	0.003	0.000	0.001	0.088
Weighted MVPA	0.000	0.000	0.065	1.451	0.147	0.000	0.000	0.075
Finding meeting and enjoyment in activities								
Cognitive impairment	−0.055	0.005	−0.504	−11.75	< 0.001	−0.065	−0.046	−0.495
Resistance PA	0.001	0.000	0.092	1.96	0.051	0.000	0.001	0.013
Weighted MVPA	−8.290e-5	0.000	−0.038	−0.813	0.417	0.000	0.000	−0.040
Relational difficulties								
Cognitive impairment	−0.059	0.004	−0.606	−15.34	< 0.001	−0.066	−0.051	−0.593
Resistance PA	0.001	0.000	0.128	2.96	0.003	0.000	0.001	0.037
Weighted MVPA	−6.412e-5	0.000	−0.033	−0.772	0.440	0.000	0.000	−0.029
Occupational/vocational functioning								
Cognitive impairment	−0.053	0.004	−0.566	−13.83	< 0.001	−0.061	−0.046	−0.554
Resistance PA	0.001	0.000	0.107	2.39	0.017	0.000	0.001	0.032
Weighted MVPA	−1.640e-5	0.000	−0.009	−0.196	0.845	0.000	0.000	−0.010
Psychological challenges								
Cognitive impairment	−0.067	0.004	−0.603	−15.19	< 0.001	−0.076	−0.059	−0.587
Resistance PA	0.001	0.000	0.106	2.45	0.015	0.000	0.001	0.041
Weighted MVPA	6.054e-5	0.000	0.027	0.630	0.529	0.000	0.000	0.023
Verbal communication challenges								
Cognitive impairment	−0.058	0.004	−0.568	−13.86	< 0.001	−0.066	−0.050	−0.559
Resistance PA	0.000	0.000	0.057	1.27	0.205	0.000	0.001	−0.008
Weighted MVPA	3.499e-5	0.000	0.017	0.386	0.700	0.000	0.000	−0.004
Social functioning and withdrawal								
Cognitive impairment	−0.063	0.005	−0.556	−13.47	< 0.001	−0.072	−0.054	−0.540
Resistance PA	0.001	0.000	0.117	2.60	0.010	0.000	0.001	0.052
Weighted MVPA	2.829e-5	0.000	0.013	0.280	0.779	0.000	0.000	0.017
Informational needs								
Cognitive impairment	−0.061	0.005	−0.558	−13.57	< 0.001	−0.070	−0.052	−0.538
Resistance PA	0.001	0.000	0.133	2.96	0.003	0.000	0.001	0.079
Weighted MVPA	8.858e-5	0.000	0.040	0.907	0.365	0.000	0.000	0.051

*PA* physical activity, *MVPA* moderate‑to‑vigorous physical activity

#### Executing regular activities

##### Difficulties

The final model accounting for perceived CRCI-related difficulties in executing regular activities was significant (*R*^2^ = 0.478, *F*_(3, 414)_ = 126.2, *p* < 0.001), with the predictors accounting for a collective 47.8% of variance in the criterion. Physical activity collectively accounted for an additional 1.2% of variance in perceived CRCI-related difficulties in executing regular activities (*R*^2^ change = 0.012, *F* change_(3, 414)_ = 4.68, adjusted *p* = 0.03) above what was contributed by perceived cognitive functioning. However, only resistance-based physical activity weekly duration (0.88%, *p* = 0.008), and not weighted MVPA, accounted for a significant amount of unique variance in perceived CRCI-related difficulties in executing regular activities, with greater resistance-based physical activity weekly duration associated with greater perceived difficulties in the semi-partialled association.

##### Unmet needs

The final model accounting for perceived CRCI-related unmet needs in executing regular activities was significant (*R*^2^ = 0.315, *F*_(3, 414)_ = 63.3, *p* < 0.001), with the predictors accounting for a collective 31.5% of variance in the criterion. Physical activity collectively accounted for an additional 2.8% of variance in perceived CRCI-related unmet needs in executing regular activities (*R*^2^ change = 0.028, *F* change_(3, 414)_ = 8.48, adjusted *p* = 0.01) above what was contributed by perceived cognitive functioning. Although once again, resistance-based physical activity weekly duration (1.2%, *p* = 0.003) and not weighted MVPA accounted for a significant amount of unique variance in perceived CRCI-related unmet needs in executing regular activities, with greater resistance-based physical activity weekly duration associated with greater levels of perceived unmet needs in the semi-partialled association.

#### Finding meaning and enjoyment in activities

##### Difficulties

The final model accounting for perceived CRCI-related difficulties in finding meaning and enjoyment in activities was significant (*R*^2^ = 0.232, *F*_(3, 414)_ = 41.8, *p* < 0.001), with the predictors accounting for a collective 23.2% of variance in the criterion. However, physical activity did not account for a significant amount of additional variance in perceived CRCI-related difficulties in finding meaning and enjoyment in activities (*R*^2^ change = 0.003, *F* change_(3, 414)_ = 0.786, adjusted *p* = 0.52) above what was contributed by perceived cognitive functioning.

##### Unmet needs

The final model accounting for perceived CRCI-related finding meaning and enjoyment in activities unmet needs was significant (*R*^2^ = 0.252, *F*_(3, 414)_ = 46.5, *p* < 0.001), with the predictors accounting for a collective 25.2% of variance in the criterion. However, as per the difficulties indices, physical activity did not account for a significant amount of additional variance in perceived CRCI-related finding meaning and enjoyment in activities unmet needs (*R*^2^ change = 0.007, *F* change_(3, 414)_ = 1.92, *p* = 0.22) above what was contributed by perceived cognitive functioning.

#### Relational difficulties

##### Difficulties

The final model accounting for perceived CRCI-related relational difficulties was significant (*R*^2^ = 0.351, *F*_(3, 414)_ = 74.6, *p* < 0.001), with the predictors accounting for a collective 35.1% of variance in the criterion. PA did not account for a significant amount of additional variance in perceived CRCI-related relational difficulties (*R*^2^ change = 0.004, *F* change_(3, 414)_ = 1.41, adjusted *p* = 0.327) above what was contributed by perceived cognitive functioning.

##### Unmet needs

The final model accounting for perceived CRCI-related relational difficulties unmet needs was significant (*R*^2^ = 0.365, *F*_(3, 414)_ = 79.3, *p* < 0.001), with the predictors accounting for a collective 36.5% of variance in the criterion. PA collectively accounted for an additional 1.4% of variance in perceived CRCI-related relational difficulties unmet needs (*R*^2^ change = 0.014, *F* change_(3, 414)_ = 4.48, adjusted *p* = 0.03) above what was contributed by perceived cognitive functioning. Once again, resistance physical activity weekly duration (0.9%, *p* = 0.003), and not weighted MVPA, accounted for a significant amount of unique variance in perceived CRCI-related relational difficulties, unmet needs, with greater resistance physical activity weekly duration associated with greater levels of perceived unmet needs in the semi-partialled association.

#### Occupational/vocational functioning

##### Difficulties

The final model accounting for perceived CRCI-related occupational/vocational functioning difficulties was significant (*R*^2^ = 0.314, *F*_(3, 414)_ = 63.1, *p* < 0.001), with the predictors accounting for a collective 31.4% of variance in the criterion. PA did not account for a significant amount of additional variance in perceived CRCI-related occupational/vocational functioning difficulties (*R*^2^ change = 0.011, *F* change_(3, 414)_ = 3.39, adjusted *p* = 0.08).

##### Unmet needs

The final model accounting for perceived CRCI-related occupational/vocational functioning unmet needs was significant (*R*^2^ = 0.317, *F*_(3, 414)_ = 64.1, *p* = 0.001), with the predictors accounting for a collective 31.7% of variance in the criterion. PA did not account for a significant amount of additional variance in perceived CRCI-related occupational/vocational functioning unmet needs (*R*^2^ change = 0.011, *F* change_(3, 414)_ = 3.21, adjusted *p* = 0.08) above what was contributed by perceived cognitive functioning.

#### Psychological challenges

##### Difficulties

The final model accounting for perceived CRCI-related psychological challenge difficulties was significant (*R*^2^ = 0.387, *F*_(3, 414)_ = 87.25, *p* < 0.001), with the predictors accounting for a collective 38.7% of variance in the criterion. Physical activity did not account for a significant amount of additional variance in perceived CRCI-related psychological challenge difficulties (*R*^2^ change = 0.008, *F* change_(3, 414)_ = 2.70, adjusted *p* = 0.121) above what was contributed by perceived cognitive functioning.

##### Unmet needs

The final model accounting for perceived CRCI-related psychological challenge unmet needs was significant (*R*^2^ = 0.356, *F*_(3, 414)_ = 39.91, *p* < 0.001), with the predictors accounting for a collective 35.6% of variance in the criterion. Physical activity collectively accounted for an additional 1.4% of variance in perceived CRCI-related psychological challenge unmet needs (*R*^2^ change = 0.014, *F* change_(3, 414)_ = 4.55, adjusted *p* = 0.03) above what was contributed by perceived cognitive functioning. Resistance-based physical activity weekly duration (0.92%, *p* = 0.015), and not weighted MVPA, accounted for a significant amount of unique variance in perceived CRCI-related psychological challenge unmet needs, with greater resistance-based physical activity duration associated with greater levels of unmet needs in the semi-partialled association.

#### Verbal communication challenges

##### Difficulties

The final model accounting for perceived CRCI-related verbal communication challenge difficulties was significant (*R*^2^ = 0.322, *F*_(3, 414)_ = 65.47, *p* < 0.001), with the predictors accounting for a collective 32.2% of variance in the criterion. Physical activity did not account for a significant amount of additional variance in perceived CRCI-related verbal communication challenge difficulties (*R*^2^ change = 0.001, *F* change_(3, 414)_ = 0.42, *p* = 0.65) above what was contributed by perceived cognitive functioning.

##### Unmet needs

The final model accounting for perceived CRCI-related verbal communication challenges unmet needs was significant (*R*^2^ = 0.317, *F*_(3, 414)_ = 29.49, adjusted *p* = 0.001), with the predictors accounting for a collective 31.7% of variance in the criterion. Physical activity did not account for a significant amount of additional variance in perceived CRCI-related verbal communication challenge unmet needs (*R*^2^ change = 0.004, *F* change_(3, 414)_ = 1.28, *p* = 0.35) above what was contributed by perceived cognitive functioning.

#### Social functioning and withdrawal

##### Difficulties

The final model accounting for perceived CRCI-related social functioning and withdrawal difficulties was significant (*R*^2^ = 0.302, *F*_(3, 414)_ = 59.72, *p* < 0.001), with the predictors accounting for a collective 30.2% of variance in the criterion. Physical activity did not account for a significant amount of additional variance in perceived CRCI-related social functioning and withdrawal difficulties (*R*^2^ change = 0.002, *F* change_(3, 414)_ = 0.78, adjusted *p* = 0.52) above what was contributed by perceived cognitive functioning.

##### Unmet needs

The final model accounting for perceived CRCI-related social functioning and withdrawal unmet needs was significant (*R*^2^ = 0.307, *F*_(3, 414)_ = 34.81, *p* < 0.001), with the predictors accounting for a collective 30.7% of variance in the criterion. Physical activity collectively accounted for an additional 1.5% of variance in perceived CRCI-related social functioning and withdrawal unmet needs (*R*^2^ change = 0.015, *F* change_(3, 414)_ = 4.43, adjusted *p* = 0.03) above what was contributed by perceived cognitive functioning. Resistance-based physical activity weekly duration (1.1%, *p* = 0.010), and not MVPA, accounted for a significant amount of unique variance in perceived CRCI-related social functioning and withdrawal unmet needs, with greater resistance-based physical activity duration associated with greater levels of unmet needs in the semi-partialled association.

#### Informational needs

##### Difficulties

The final model accounting for perceived CRCI-related informational need difficulties was significant (*R*^2^ = 0.289, *F*_(3, 414)_ = 56.06, *p* < 0.001), with the predictors accounting for a collective 28.9% of variance in the criterion. Physical activity collectively accounted for an additional 5.9% of variance in perceived CRCI-related social functioning and withdrawal unmet needs (*R*^2^ change = 0.059, *F* change_(3, 414)_ = 17.10, adjusted *p* = 0.01) above what was contributed by perceived cognitive functioning. Resistance-based physical activity weekly duration (3.1%, *p* < 0.001), and weighted MVPA (0.69%, *p* = 0.045), accounted for a significant amount of unique variance in perceived CRCI-related informational need difficulties, with greater resistance-based and aerobic-based physical activity weekly duration associated with greater levels of difficulties in the semi-partialled associations.

##### Unmet needs

The final model accounting for perceived CRCI-related informational unmet needs was significant (*R*^2^ = 0.312, *F*_(3, 414)_ = 62.66, *p* < 0.001), with the predictors accounting for a collective 31.2% of variance in the criterion. Physical activity collectively accounted for an additional 2.3% of variance in perceived CRCI-related informational unmet needs (*R*^2^ change = 0.023, *F* change_(3, 414)_ = 6.98, adjusted *p* = 0.01) above what was contributed by perceived cognitive functioning. Resistance physical activity weekly duration (1.4%, *p* = 0.003), but not weighted MVPA, accounted for a significant amount of unique variance in perceived CRCI-related informational unmet needs, with greater resistance-based physical activity weekly duration associated with greater levels of unmet needs in the semi-partialled association.

## Discussion

This study examined associations between physical activity, perceived cognitive functioning, and CRCI-related difficulties and unmet needs, and whether physical activity explained additional variance in CRCI-related difficulties and unmet needs beyond perceived cognitive functioning. Weekly physical activity duration across light and vigorous aerobic intensities, as well as resistance physical activity weekly duration, was associated with better perceived cognitive functioning. However, weekly duration of moderate-intensity aerobic physical activity and weighted MVPA were not significantly associated with perceived cognitive functioning. Associations between light-and vigorous-intensity aerobic and resistance-based physical activity and better perceived cognitive functioning align strongly with the body of interventional research literature examining the efficacy of physical activity on CRCI [27, 28, 56]. However, the lack of associations for moderate-intensity aerobic activity and weighted MVPA is in contrast to the majority of the interventional literature [27, 28, 56], though it aligns with the mixed and equivocal literature on physical activity on cognitive functioning in cancer in observational research [27, 57]. Brunet and Sharma’s scoping review [57] found that only 30.8% of observational research examining the association between physical activity and perceived cognitive functioning found significant positive associations. This may suggest that the observational research, the majority of which has been cross-sectional, may not capture the dynamic relationship between physical activity and perceived cognitive functioning in cancer due to factors such as the *improvements* in perceived cognitive functioning from participation in physical activity, as well as the potential for physical activity to mitigate significant perceived cognitive decline [27, 28, 56].

At the bivariate level the associations between physical activity and perceived CRCI-related difficulties were mixed. Greater participation in light-intensity aerobic physical activity was associated with fewer perceived CRCI-related psychological difficulties, whereas greater participation in vigorous-intensity aerobic physical activity was associated with fewer perceived CRCI-related verbal communication difficulties. In contrast, greater participation in light-and vigorous-intensity aerobic physical activity, as well as resistance-based physical activity, was associated with greater perceived CRCI-related informational needs difficulties. Further, no associations were found between physical activity and perceived CRCI-related unmet needs. However, when partialling out the effect of perceived cognitive functioning on perceived CRCI-related difficulties and unmet needs, clearer patterns of effect emerged – with greater engagement in physical activity associated with greater perceived CRCI-related difficulties and unmet needs in several domains. After controlling for perceived cognitive functioning, greater engagement in physical activity was associated with higher levels of perceived CRCI-related difficulties in executing regular activities and greater CRCI-related informational needs, with resistance-based physical activity contributing most of the predictive utility. In line with this, after controlling for perceived cognitive functioning, greater engagement in physical activity, in particular resistance-based physical activity, was associated with greater levels of perceived CRCI-related unmet needs in five out of the eight MASCC COG-IMPACT subscales. Aligning with other literature exploring psychosocial, demographic, and clinical predictors of perceived CRCI-related difficulties and unmet needs [4, 34], these findings suggest physical activity may be associated with a range of perceived CRCI-related difficulties and unmet needs through other mechanisms beyond cognitive functioning. Greater physical activity engagement has been associated with higher health literacy, health information seeking, self-management capacity, and awareness of bodily and cognitive changes [58–60]. Consequently, while physical activity may reduce CRCI severity, more active cancer survivors may also be more attuned to CRCI-related challenges and more likely to recognise and report associated difficulties and unmet supportive care needs. In addition, higher levels of physical activity often coincide with engagement in cognitively, physically, and socially demanding roles and activities where CRCI-related difficulties and unmet needs may be more salient [34, 61, 62].

As effect sizes were generally small, and the additional variance explained by physical activity beyond perceived cognitive functioning was modest, these findings should be interpreted as indicating small but potentially meaningful associations rather than strong clinical effects. However, these findings underscore the importance of conceptualising the role of physical activity in CRCI within a holistic and person-centred survivorship framework, rather than focusing solely on cognitive symptom severity. While physical activity is a promising strategy to improve perceived cognitive functioning for cancer survivors, its associations with CRCI-related difficulties and unmet supportive care needs are more complex and may reflect broader patterns of health literacy, information seeking, role engagement, and participation. Collectively, these findings support physical activity as a promising strategy for addressing CRCI while highlighting that improvements in perceived cognitive functioning may not necessarily correspond with lower levels of CRCI-related difficulties and unmet needs. Consequently, physical activity interventions for CRCI should be embedded within holistic survivorship care models that incorporate routine assessment and management of these broader functional and supportive care concerns.

### Strengths, limitations, and directions for future research

This study had several notable strengths, including the use of a large sample of cancer survivors with broad international representation across key clinical and demographic characteristics, such as age, sex, ethnicity, country of residence, cancer type, and treatment exposure. Future research should examine these relationships within more homogeneous clinical subgroups. This study also applied methods, statistical techniques, and reporting aligned with best-practice guidelines [41, 63, 64], facilitating robust interpretation within the constraints of a cross-sectional study design. This study, however, also had some key limitations. This sample is limited to only cancer survivors who were undergoing cancer treatment with curative-intent. While this is the population that most severely experiences CRCI [2, 65, 66], future studies should extend this work by examining cancer survivors at other points along the cancer care continuum. Furthermore, while the diversity of the sample strengthens generalisability, it may preclude subgroup-specific inferences. Future research should examine these relationships within more homogeneous clinical subgroups. All data-points were also self-reported, which increases the potential for error, results reflecting common-method variance, and mitigates the ability to examine objective measures of physical activity and cognitive function. In addition, the measures utilised different recall periods (e.g., previous seven days versus previous month), which may introduce some temporal mismatch when interpreting the observed associations. Future research may utilise medical records and include objective assessments and multi-method approaches. In addition, comprehensive clinical and psychosocial data were not collected, including indicators such as body mass index, blood pressure, comorbid conditions, health literacy, and social engagement. These unmeasured factors may act as important moderators or mediators of the relationships between physical activity, perceived cognitive functioning, and perceived CRCI-related difficulties and unmet supportive care needs [8]. Therefore, future research should collect a broader collection of clinical and psychosocial variables. It remains possible that cancer type- and treatment-specific factors influence the observed associations- in particular neurological cancers and treatments. However, these relationships could not be examined in the present study due to numerous small cancer subgroups and non-mutually exclusive treatment exposures. Most participants (68.4%) also met physical activity guidelines, which strongly exceeds previous studies (i.e., ~27.6%) [67], as well as a relatively young mean age (39.4 years), potentially reflecting self-selection bias and an overrepresentation of more health-engaged survivors who may be more likely to identify and report CRCI-related difficulties and unmet needs. Finally, this study employed a cross-sectional design, which is appropriate for establishing initial evidence, but does not permit examination of temporal sequencing or causal relationships. Future studies should prioritise longitudinal designs.

## Conclusion

Physical activity was associated with better perceived cognitive functioning among cancer survivors reporting CRCI; however, these associations did not consistently correspond with lower levels of CRCI-related difficulties and unmet needs. Accordingly, physical activity should be considered within broader survivorship care models that address both cognitive functioning and the functional and supportive care challenges associated with CRCI. Given the clinical heterogeneity of the sample, these findings should not be assumed to apply equally across all cancer types or treatment groups, and future research should examine these relationships within more homogeneous cancer populations. Further, given the cross-sectional nature of this study, the observed relationships should be interpreted as associations and no causal conclusions can be drawn at this stage.

## Supplementary Information

Below is the link to the electronic supplementary material.

ESM 1 (PDF 214 KB)

## Data Availability

Data may be made available upon reasonable request of the corresponding author and to the satisfaction of the MREC.
