# Supplementary material for: Physical activity and cancer-related cognitive impairment: Is physical activity associated with perceived difficulties and unmet needs?
Source: Support Care Cancer. 2026 Nov 7;34(12):1202. doi: 10.1007/s00520-026-11429-9 (PMC13744451; doi:10.1007/s00520-026-11429-9)
Supplement: Supplementary file 1 [file 520_2026_11429_MOESM1_ESM.pdf]

STROBE Statement—checklist of items that should be included in reports of observational studies

	Item No.	Recommendation	Page No.	Relevant text from manuscript
**Title and abstract**	1	(*a*) Indicate the study's design with a commonly used term in the title or the abstract	1	"In this cross-sectional study, adult cancer survivors receiving curative-intent treatment (n=418)."
		(*b*) Provide in the abstract an informative and balanced summary of what was done and what was found	1	Entire structured abstract (Purpose, Methods, Results, Conclusions).
**Introduction**				
Background/rationale	2	Explain the scientific background and rationale for the investigation being reported	1-2	"Physical activity is a cost-effective, feasible, and accessible approach to mitigate CRCI..." and rationale explaining uncertainty regarding CRCI-related difficulties and unmet needs.
Objectives	3	State specific objectives, including any prespecified hypotheses	2	"Accordingly, the aims of this study were to (1) examine associations between physical activity, perceived cognitive functioning, and perceived CRCI-related difficulties and unmet supportive care needs... and (2) assess if physical activity explains additional variance..."
**Methods**				
Study design	4	Present key elements of study design early in the paper	2	"A cross-sectional self-report

				study design was used”
Setting	5	Describe the setting, locations, and relevant dates, including periods of recruitment, exposure, follow-up, and data collection	3	"Participants were recruited via Prolific..." and study procedures described under Recruitment and Methods.
Participants	6	(*a*) *Cohort study*—Give the eligibility criteria, and the sources and methods of selection of participants. Describe methods of follow-up<br>*Case-control study*—Give the eligibility criteria, and the sources and methods of case ascertainment and control selection. Give the rationale for the choice of cases and controls<br>*Cross-sectional study*—Give the eligibility criteria, and the sources and methods of selection of participants	3	Eligibility criteria: "Participants were adult cancer survivors, defined as..." plus exclusion criterion and recruitment via Prolific.
		(*b*) *Cohort study*—For matched studies, give matching criteria and number of exposed and unexposed<br>*Case-control study*—For matched studies, give matching criteria and the number of controls per case	N/A	N/A
Variables	7	Clearly define all outcomes, exposures, predictors, potential confounders, and effect modifiers. Give diagnostic criteria, if applicable	3	Detailed definitions of MASCC COG-IMPACT, PROMIS-Cog, GSLTPAQ, weighted MVPA, CRCI difficulties, unmet needs and cognitive functioning
Data sources/ measurement	8*	For each variable of interest, give sources of data and details of methods of assessment (measurement). Describe comparability of assessment methods if there is more than one group	3-4	Measurement descriptions for MASCC COG-IMPACT, PROMIS-Cog, and adapted GSLTPAQ
Bias	9	Describe any efforts to address potential sources of bias	3, 12-13	Recruitment based on self-identification of CRCI; attention checks; limitations discuss self-report measures, recall mismatch, self-selection bias.
Study size	10	Explain how the study size was arrived at	4	"Although an a priori sample size calculation was not

	performed, the final sample of 418 participants substantially exceeded..."

Continued on next page

Quantitative variables	11	Explain how quantitative variables were handled in the analyses. If applicable, describe which groupings were chosen and why	3-4	Weighted MVPA calculation, PROMIS-Cog scoring, MASCC COG-IMPACT scoring and cut-offs, regression analyses.
Statistical methods	12	(*a*) Describe all statistical methods, including those used to control for confounding	3-4	Correlations, hierarchical regressions, FDR corrections, interpretation of effect sizes.
		(*b*) Describe any methods used to examine subgroups and interactions	N/A	N/A
		(*c*) Explain how missing data were addressed	N/A	N/A
		(*d*) *Cohort study*—If applicable, explain how loss to follow-up was addressed<br>*Case-control study*—If applicable, explain how matching of cases and controls was addressed<br>*Cross-sectional study*—If applicable, describe analytical methods taking account of sampling strategy	3-4	Recruitment through Prolific and participant eligibility criteria described.
		(*e*) Describe any sensitivity analyses	N/A	N/A
**Results**				
Participants	13*	(a) Report numbers of individuals at each stage of study—eg numbers potentially eligible, examined for eligibility, confirmed eligible, included in the study, completing follow-up, and analysed	4	"Four hundred and twenty-two (n=422) cancer survivors completed the survey, with three removed due to failing 50% or more of the four attention check items. Thus, a total sample of 418..."
		(b) Give reasons for non-participation at each stage	4	Three participants removed for attention check failures.
		(c) Consider use of a flow diagram	N/A	N/A
Descriptive data	14*	(a) Give characteristics of study participants (eg demographic, clinical, social) and information on exposures and potential confounders	4-6; Tables 1-2	Sample characteristics and descriptive statistics reported in Tables 1 and 2.
		(b) Indicate number of participants with missing data for each variable of interest	N/A	N/A
		(c) *Cohort study*—Summarise follow-up time (eg, average and total amount)	N/A	N/A
Outcome data	15*	*Cohort study*—Report numbers of outcome events or summary measures over time	N/A	N/A
		Case-control study—Report numbers in each exposure category, or summary measures of exposure	N/A	N/A

		Cross-sectional study—Report numbers of outcome events or summary measures	4-3; Tables 2-5	Descriptive statistics, correlations, and regression outcomes reported
Main results	16	(*a*) Give unadjusted estimates and, if applicable, confounder-adjusted estimates and their precision (eg, 95% confidence interval). Make clear which confounders were adjusted for and why they were included	Tables 3-5	Correlations, regression coefficients, R^2 values, p-values, FDR-adjusted analyses.
		(*b*) Report category boundaries when continuous variables were categorized	Table 2	PROMIS-Cog clinical cut-off, MASCC unmet need categories, MVPA guideline classification ≥150 mins/week.
		(*c*) If relevant, consider translating estimates of relative risk into absolute risk for a meaningful time period	N/A	N/A

Continued on next page

Other analyses	17	Report other analyses done—eg analyses of subgroups and interactions, and sensitivity analyses	3-4; 6-11	Benjamini-Hochberg FDR analyses and hierarchical regressions.
**Discussion**				
Key results	18	Summarise key results with reference to study objectives	11-12	Opening discussion paragraph summarising findings.
Limitations	19	Discuss limitations of the study, taking into account sources of potential bias or imprecision. Discuss both direction and magnitude of any potential bias	12-13	Entire "Strengths, Limitations, and Directions for Future Research" section
Interpretation	20	Give a cautious overall interpretation of results considering objectives, limitations, multiplicity of analyses, results from similar studies, and other relevant evidence	11-13	Discussion section interpreting findings in relation to prior literature and study aims
Generalisability	21	Discuss the generalisability (external validity) of the study results	11-13	Discussion of broad international sample, diversity, subgroup limitations and survivorship populations.
**Other information**				
Funding	22	Give the source of funding and the role of the funders for the present study and, if applicable, for the original study on which the present article is based	13	"This project was funded by a UTS Faculty of Health Seeds Funding grant..."

*Give information separately for cases and controls in case-control studies and, if applicable, for exposed and unexposed groups in cohort and cross-sectional studies.

Note: An Explanation and Elaboration article discusses each checklist item and gives methodological background and published examples of transparent reporting. The STROBE checklist is best used in conjunction with this article (freely available on the Web sites of PLoS Medicine at http://www.plosmedicine.org/, Annals of Internal Medicine at http://www.annals.org/, and Epidemiology at http://www.epidem.com/). Information on the STROBE Initiative is available at www.strobe-statement.org.
